# Worry Modifies the Relationship between Locus Coeruleus Activity and Emotional Mnemonic Discrimination

**DOI:** 10.3390/brainsci12030381

**Published:** 2022-03-12

**Authors:** Linda H. G. Pagen, Benedikt A. Poser, Martin P. J. van Boxtel, Nikos Priovoulos, Roy W. E. van Hooren, Frans R. J. Verhey, Heidi I. L. Jacobs

**Affiliations:** 1 Alzheimer Centre Limburg, School for Mental Health and Neuroscience, Faculty of Health, Medicine and Life Sciences, Maastricht University, 6200 MD Maastricht, The Netherlands; linda.pagen@maastrichtuniversity.nl (L.H.G.P.); martin.vanboxtel@maastrichtuniversity.nl (M.P.J.v.B.); nikos.priovoulos@maastrichtuniversity.nl (N.P.); roy.vanhooren@maastrichtuniversity.nl (R.W.E.v.H.); f.verhey@maastrichtuniversity.nl (F.R.J.V.); 2 Centre for Integrative Neuroscience, School for Mental Health and Neuroscience, Faculty of Psychology and Neuroscience, Maastricht University, 6200 MD Maastricht, The Netherlands; 3 Department of Cognitive Neuroscience, Faculty of Psychology and Neuroscience, Maastricht University, 6200 MD Maastricht, The Netherlands; benedikt.poser@maastrichtuniversity.nl; 4 Gordon Center for Medical Imaging, Department of Radiology, Massachusetts General Hospital, Harvard Medical School, Boston, MA 02114, USA

**Keywords:** worry, emotional mnemonic discrimination, locus coeruleus, aging

## Abstract

Background: The locus coeruleus (LC) plays a critical role in modulating emotional memory performance via widespread connections to the medial temporal lobe (MTL). Interestingly, both the LC and MTL are affected during aging. Therefore, we aimed to investigate whether worry during cognitive aging changes the relationship between memory performance and the neural activity patterns during an emotional memory task. Methods: Twenty-eight participants aged 60–83 years from the Maastricht Aging study conducted an emotional mnemonic discrimination task during a 7T fMRI-scan. We performed a robust multiple linear regression to examine the association between worry and mnemonic memory performance under different levels of arousal. Subsequently, we examined if worry modifies the relationship between neuronal activity and mnemonic memory performance. Results: We observed that under low arousal, only participants with low compared to high levels of worry benefitted from additional LC activity. Under high arousal, additional LC activity was associated with lower mnemonic memory performance. Conclusion: Our results suggest there might be an optimal involvement of the NA-system for optimal memory discrimination performance, as we observed that under low levels of worry and with lower levels of arousal, higher LC activity might be needed to achieve similar levels of optimal memory performance as achieved under higher arousal when LC activity remained lower.

## 1. Introduction

Ample studies demonstrated that negative emotional arousal and the associated release of noradrenaline (NA) enhance memory performance [1,2,3,4]. Central NA stems mainly from the locus coeruleus (LC), a small nucleus in the brainstem [5,6]. The LC is believed to play a critical role in modulating memory performance through its widespread connections, and in particular through the effect of NA on adrenergic receptors in the medial temporal lobe [7,8,9,10]. Interestingly, both the LC and the medial temporal lobe (MTL) play an important role in both memory performance and emotional regulation, two processes that are well known to be affected during aging [2,11,12,13]. Therefore, these regions could play an important role in emotional memory performance within an older population.

Emotional mnemonic discrimination, a memory process that relies on both the MTL structures and the LC, is well suited to investigate the modulation of arousal on memory [4,14,15]. Mnemonic discrimination refers to the ability to discriminate highly similar items and can be used as a proxy for hippocampal pattern separation processes [16]. This is the ability in which similar representations are stored in a distinct, non-overlapping manner. Within the MTL, mnemonic discrimination relies on the dentate gyrus (DG) and the cornu ammonis (CA) 3 of the hippocampus [16]. Age-related changes in activity in the DG and CA3 have been related to lower mnemonic discrimination performance [17]. For younger adults, DG/CA3 activity facilitates correct mnemonic discrimination, especially under high arousal [14]. While in older adults there seems to be a delicate balance in which high arousal can still promote performance but hyperactivation of the DG/CA3 independent of arousal leads to lower mnemonic discrimination performance [18]. This hyperactivation has been suggested to be an aberrant process reflecting age-related neural network breakdown. Seminal work from Harley [13,19] demonstrated in rodents that activation of the modulatory projections from the LC to the DG and the amygdala lead to long-lasting enhancement of cell firing in the DG and perforant pathway, contributing to enhanced hippocampus-dependent memory performance [2,12]. In imaging studies of older individuals, an intact structural integrity of the LC was associated with better memory performance [20], specifically for high-arousing events [21]. Beyond its critical role in learning and emotion, the LC is also vulnerable to the earliest pathologic processes associated with Alzheimer’s disease (AD) [22,23,24]. These early changes in the LC have been reported to occur two to three decades before the first clinical symptoms can be detected. At this stage, individuals often report worries about their memory functioning, and as such, they may be at higher risk for memory decline [25,26,27,28,29,30,31]. Interestingly, individuals reporting a higher level of worry about memory functioning demonstrated a lower capacity for emotion regulation in response to a memory challenge [32], which was reflected in a higher negative affect and lower heart rate variability, an indirect measure of less well-regulated autonomic nervous system activity. Together these studies suggest that examining the functioning of the LC during an emotional memory task may reveal important underlying mechanistic information of risk of memory decline in individuals with greater worries. Given that the LC is affected early in life and that the emotional mnemonic discrimination task is also sensitive to mid-life memory changes, the results of this study can contribute to identifying those individuals with worries who are also at the greatest risk of cognitive decline.

Therefore, the current study aimed to investigate whether the level of reported worry changes the relationship between memory performance and the neural activity patterns of the LC and MTL regions during an emotional memory task in older individuals. We hypothesized a higher mnemonic discrimination score for high arousal trials compared to low arousal trials. Additionally, we expect that worry will be negatively associated with mnemonic discrimination performance. Furthermore, consistent with previous studies [33], we expected that activity in the CA3 and DG is associated with a higher mnemonic discrimination performance. Additionally, given the role of the LC and the amygdala in emotional regulation, we expected higher activity in the amygdala and LC to be associated with better performance under high arousal. Finally, we expected that worries would modify the relationship between arousal and mnemonic discrimination performance: we expected that higher levels of cognitive worry would be associated with lower mnemonic discrimination performance and with higher activation of the CA3, DG, amygdala, and LC for high arousal trials.

## 2. Materials and Methods

### 2.1. Participants

Twenty-eight cognitively healthy participants from the longitudinal Maastricht Aging Study (MAAS) participated in this study, with a mean age of 68.2 years (SD = 5.0), of whom 46.4% were female. The MAAS is a population study investigating the determinants of cognitive aging [34], of which 12-year follow-up data is currently available. At baseline, 1823 participants were enrolled in MAAS [35]. The current study included participants who were native Dutch speakers, right-handed, and at the time of our data collection, between 60 and 85 years of age. Furthermore, to ensure a broad range in the reported level of worries about memory functioning, participants were included based on previous worry scores over time. To this end, we performed a latent class analysis (for details see Supplementary Materials File S1) and sampled participants from the stable high worry and low worry classes over time, and matched them for age and sex (Figure S1). Exclusion criteria were: reduced vision (correction > +/−6.0 diopter), psychoactive medication use, alcohol or drug abuse, below average neuropsychological test results (based on normative data, adjusted for age, sex, and education), past or present psychiatric or neurological disorders, major vascular disorders (e.g., stroke, heart attack), pacemaker use, and contraindications for MR scanning. Informed consent was obtained from all subjects involved in the study and the study was approved by the local Ethics Review Board and written informed consent was obtained from all participants following the Declaration of Helsinki [36].

### 2.2. Measurements and Tests

#### 2.2.1. Neuropsychological Testing and Questionnaires

Participants underwent a comprehensive neuropsychological test battery, including the Mini-Mental State Exam (MMSE; global cognitive functioning) [37], memory performance using the Verbal Learning Test (VLT; [38]), including both the verbal learning and the delayed-recall trial. Information processing speed and executive functioning were measured using the Letter-Digit-Substitution Test (LDST) [39], the Stroop Color and Word test [40], and the Concept Shifting Task (CST) [41]. Symptoms of depression were measured with the Hamilton Depression Rating Scale (HDRS) [42].

#### 2.2.2. Worry Questionnaire

Within the current study, we defined worry as the cognitive component of anxiety, reflecting people negative thoughts on how they perceive their memory performance. Subjective worry about memory functioning was measured with a subscale of the Metamemory In Adulthood (MIA) questionnaire [43]. The MIA asks participants to reflect on a 5-point Likert scale to statements about their memory functioning. Worry was measured with the anxiety subscale which specifically questions perceived feelings about stress and worry related to their memory performance. For the remainder of this manuscript, the term worry refers to the MIA-anxiety score. The MIA-anxiety subscale correlated positively to the depression scores (r_s_ = 0.39, *p* = 0.04; Figure S2), demonstrating that they capture related, but different, concepts.

#### 2.2.3. Emotional Mnemonic Discrimination Memory Task

**Figure 1:**
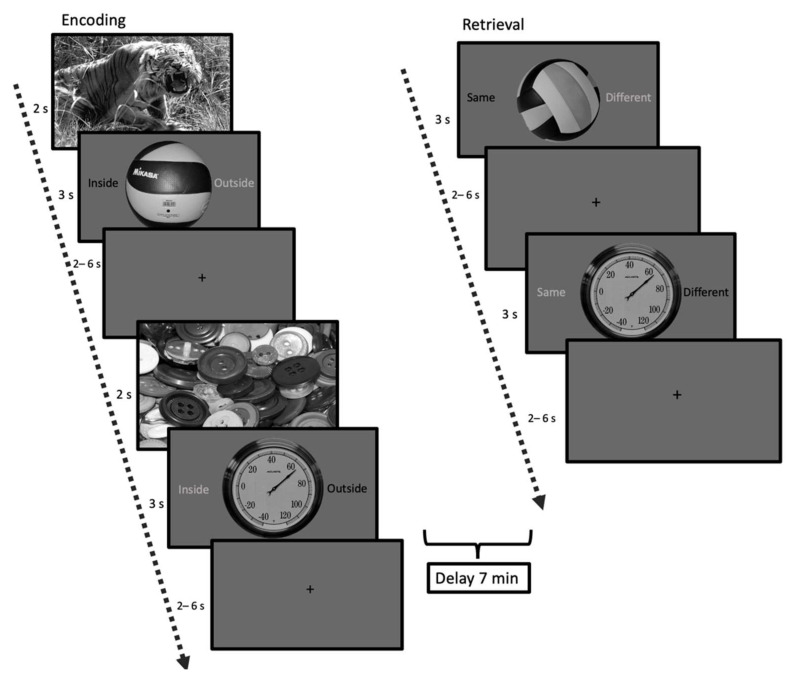
Schematic representation of the mnemonic discrimination task. Encoding consisted of 100 everyday objects shown in either a high (e.g., tiger) or low (e.g., buttons) arousing context. After a 7-min resting-state period participants were asked to indicate if the item was the same or different as seen during the encoding phase (retrieval phase).

Based on the memory task by Sterpenich and D’Argembeau [44], we developed an emotional mnemonic discrimination memory task. To this end, 100 emotional images were selected from the International Affective Picture System (IAPS) [45], divided over 50 high-arousing negatively valanced (HA) and 50 low-arousing neutral valanced (LA) pictures (Figure 1). These pictures formed the emotional context and were displayed for two seconds before the target stimulus. These images have been thoroughly validated to consistently elicit a specific emotional response (94% consistency) [45]. We selected 100 pictures of neutral daily objects as target stimuli from the mnemonic discrimination task by Kim and Yassa [46]. These objects were displayed for 3 s, and to ensure attention to the displayed objects, participants were asked to indicate if they identified the object as an indoor or outdoor object via a button press. In between the trials, we showed a fixation cross with a jitter of an average length of 3 s (range: 2–6 s, optimized with optseq [47]). All pictures were grey-scaled and luminance corrected with the use of the Matlab-based SHINE-toolbox [48]. Following this learning phase, participants underwent a 7-min resting-state scan (here termed “consolidation period”) during which participants viewed a fixation cross. Finally, participants engaged in the retrieval task. This consisted of 150 object pictures, of which participants had to indicate with a button press if the object was “same”, i.e., an exact repetition of the object seen during encoding, or “different”. This could be either a lure object which is perceptually similar, but not identical to an object seen during encoding (for an example see Figure 1) or a completely new picture not presented during encoding (50 old items, 50 similar items, and 50 new items).

Memory performance was assessed by calculating hits, false positives (FP) (similar items answered as “same”), and false negatives (FN) (old items answered with “different”) stratified by arousal level (high vs. low). The arousal condition was determined by the IAPS picture that preceded the object. For the BOLD-modeling we only used the activity during the target events without including the context.

### 2.3. Imaging

#### 7T Imaging Parameters

MRI scans were performed using a 7T Magnetom Siemens (Siemens Healthineers, Erlangen, Germany) with a 32-receive-channel head coil (Nova Medical, Wilmington, MA, USA). First, we acquired a Magnetization Prepared 2 Rapid Acquisition Gradient Echoes (MP2RAGE) sequence [49] for whole-brain T1-weighted imaging (TR = 5000 ms, TE = 2.47 ms, flip angle = 5°/3°, voxel size = 0.7 × 0.7 × 0.7 mm^3^, number of slices = 240). To image the LC structurally at high resolution and contrast, we used an in-house developed magnetization transfer-weighted turbo flash (MT-TFL) sequence [50], consisting of a multi-shot 3D readout (TR = 538 ms, TE = 4.08 ms, flip angle = 8°, voxel size = 0.4 × 0.4 × 0.5 mm^3^, number of slices = 60) with center-out k-space sampling, preceded by 20 off-resonant Gaussian sinc pulses for MT preparation (pulse length = 5.12 ms, bandwidth = 250 Hz, B1 = 0.25 μT, 2000 Hz off-resonance). The field-of-view (FOV) was placed approximately perpendicular to the pons and covered the area between the inferior colliculus and the inferior border of the pons. A multiband gradient-echo EPI sequence [51,52] was acquired for the high resolution BOLD fMRI images (TR = 2000 ms, TE = 19 ms, isotropic voxel size = 1.25 × 1.25 ×1.25 mm^3^, number of slices = 50, multiband factor = 2, GRAPPA R = 3). To optimize the hippocampal subfield differentiation, the field of view was placed roughly perpendicular to the hippocampus and at an angle at 45^o^ to the brainstem, which tends to reduce the effect of physiological movement around the pons [53]. After each BOLD fMRI acquisition, five additional volumes were acquired with the phase encoding direction reversed to facilitate distortion correction.

### 2.4. Preprocessing of MRI Data

#### 2.4.1. Anatomical Preprocessing

The T1-weighted MP2RAGE images were processed using FreeSurfer (FS) version 6.0.0 (https://surfer.nmr.mgh.harvard.edu/ (accessed on 1 June 2021)) using the software package’s default, automated reconstruction protocol as described previously [54]. To this end, each T1-weighted image was subjected to an automated segmentation process involving intensity normalization, skull stripping, segregating left and right hemispheres, removing brainstem and cerebellum, correcting topology defects, defining the borders between grey/white matter and grey/cerebrospinal fluid, and parcellating cortical and subcortical areas. To acquire a mask of the hippocampal subfields, the hippocampal-amygdala segmentation algorithm in FS was applied, which predicts the location of subregions by using a probabilistic atlas built from a combination of manual delineations of the hippocampal formation from ultra-high resolution ex-vivo MRI scans and manual annotations of the surrounding subcortical structures (e.g., amygdala, cortex) on an independent dataset [55]. Using FS’s visualization toolbox, freeview, we visually inspected and, if necessary, edited and corrected each image for over-or under-estimation of the gray/white matter boundaries and to identify brain areas erroneously excluded during skull stripping. In addition, we checked that the hippocampal subregion mask was well positioned and that the ranking of subfield-specific volumes was consistent with the literature.

The LC mask was acquired with a study-specific high-resolution binary template. Briefly, intensity-normalized images were obtained by dividing individual MT-TFL images by the subject-specific mean intensity of a 10 × 10 voxel region-of-interest (ROI) located in the middle of the pontine tegmentum. Next, the template was built based on all individual intensity-normalized MT-TFL images using the *buildtemplateparallel* function from the Advanced Normalization Tools (ANTs) [56]. The LC was manually (RvH) delineated on the resulting template in the common space, based on voxel intensities and the known LC anatomy. This bilateral LC mask was then warped back to the individual space and applied to the individual T1-image space using the WarpImageMultiTransform and antsApplyTransforms functions to create a subject-specific anatomic proxy for the fMRI analyses. The bilateral LC ROIS were visually checked for anatomical accuracy and all ROIS were found to be accurately positioned (For an example see Figure S5).

#### 2.4.2. Preprocessing of BOLD fMRI Data

Image preprocessing of the fMRI data was performed with FSL 5.0.9 (https://fsl.fmrib.ox.ac.uk/fsl/fslwiki/ (accessed on 1 June 2021)) with standard preprocessing procedures: first, five volumes were removed to ensure that only data within the longitudinal magnetization steady-state were examined. The functional images were motion-corrected to the last image with a 6-dof transform using MCFLIRT [57]. A displacement field was calculated and applied with FSL-TOPUP by using the last 5 frames of each fMRI scan and the following reversed phase encoding direction scans to reduce the EPI-distortions. We subsequently applied slice-timing correction and correction for motion artifacts using the ICA-AROMA toolbox. Finally, the data were smoothed with a smoothing kernel of 1.5 mm FWHM, consistent with the minimum LC width.

#### 2.4.3. ROI Value Extraction

Freesurfer was performed using our own brain mask and both the T1-weighted image and T1-map (that was used as a substitute for a T2-weighted image) to improve white matter and grey matter delineation. Using the anatomical labels provided by FS, we created masks of the bilateral amygdala, CA1, CA3, DG, 4rth ventricle and the white matter in T1-space. A 6-dof boundary-based registration was calculated for the linear registration between the fMRI and T1data using FSL’s epi_reg based on the FreeSurfer segmentations. Subsequently, all the ROIs as well as our LC mask were projected to functional space with nearest-neighbor interpolation.

First-level general linear models were computed for each participant. Our main focus was on emotional mnemonic conditions, and as FN returned a limited amount of trials, we restricted our analyses to hits and FP. We created the following contrasts of parameter estimates to estimate the effect of arousal: HA Hits > LA Hits, HA FP > LA FP, LA Hits > HA Hits, and LA FP > HA FP. Additionally, the contrasts: HA Hits > HA FP, LA Hits > LA FP, HA FP > HA Hits, and LA FP > LA Hits were created to estimate the effect of mnemonic discrimination. We corrected for covariates of no interest, including white matter signal, 4th ventricle signal, as well as motion parameters and motion outliers by regressing out their temporal derivatives. Subsequently, we extracted the mean beta estimates for our ROIs from these contrasts. We averaged the mean beta estimates for all the ROIs across the left and right hemisphere, as we had no predefined lateralization hypotheses.

### 2.5. Statistical Analyses

All statistical analyses were done using R 3.5.2 [58] (http://www.R-project.org/ (accessed on 1 June 2021)).

Welch paired two-sample *t*-tests were used to examine differences in mnemonic discrimination performance between high and low arousal trials and differences in mean beta activation for every ROI for low versus high arousal trials as well as for hits versus FP.

Subsequently, to minimize the influence of possible outliers, we performed robust multiple linear regression models using the Huber-M estimator and rescaling using the predetermined MAD of the residuals for each memory performance outcome value, including HA Hits, HA FP, LA Hit, and LA FP, to examine the relationship between worry and memory performance:

1. Memory performance ~ age + sex + depression score + worry score

To verify the association between ROI activity and memory performance as well as an interaction between ROI activity and worry for all the arousal and the memory contrasts, we performed the following robust multiple linear regressions for each memory performance outcome value:

2. Memory performance ~ age + sex + worry score + ROI contrast activity
3. Memory performance ~ age + sex + worry score × ROI contrast activity

All *p*-values were two-sided, and all *p* < 0.05 are reported as significant. We corrected for multiple comparisons using the False Discovery rate (FDR)-approach [59], indicated by p_FDR_.

## 3. Results

The final study sample included a total of 23 participants with a mean age of 67.1 years (SD = 4.9), of whom 47.8% were female (Table 1). Two participants were excluded from further analyses due to faulty button presses during the memory task and for three participants the ICA revealed only components attributable to motion and were therefore excluded from further analyses.

**Figure 2:**
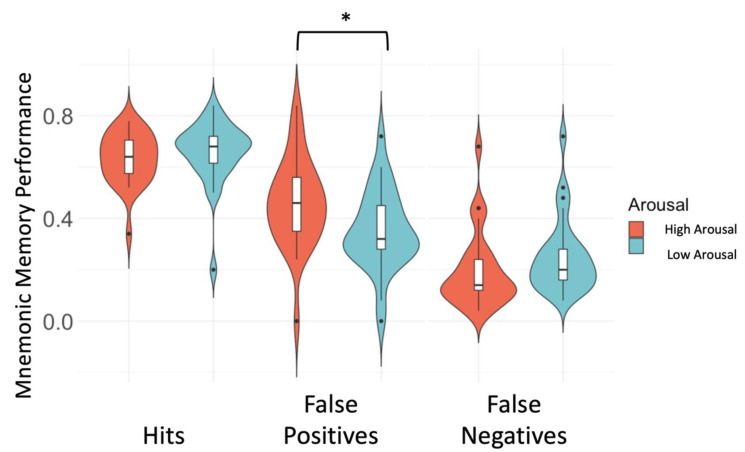
Memory performance under high and low arousal. More False Positives were observed for high arousal trials compared to low arousal trials. No differences were observed for Hits and False Negatives. Y-axis equals the mean proportion of Hits, False Positives, and False Negatives. Boxplots reflect the median and inter quartile range * *p* < 0.05.

First, the difference between high and low arousal trials on memory performance revealed significantly more FP in the HA-condition compared to the LA-condition (t(53.3) = 2.46, *p* = 0.02; Figure 2), no difference in arousal level was observed for Hits (t(51.7) = −0.80, *p* = 0.43) or FN (t(53.9) = −1.47, *p* = 0.15). We observed a trend towards a positive association between worry and Hits, for stimuli within a low arousal context (β = 0.07, t = 2.02, df = 23, *p* = 0.06), not during high arousal (β = 0.03, t = 0.71, df = 23, *p* = 0.49). There was no association between worry and FP, for either high or low arousal contexts (HA: β = −0.006, t = 0.10, df = 23, *p* = 0.92; LA: β = −0.03, t = −0.53, df = 23, *p* = 0.59). We observed no difference in low versus high arousal trials for activation in any of the ROIs during Hits (Figure S3) or FP (Figure S4).

### 3.1. High Arousal Modulates Mnemonic Discrimination Performance

**Figure 3:**
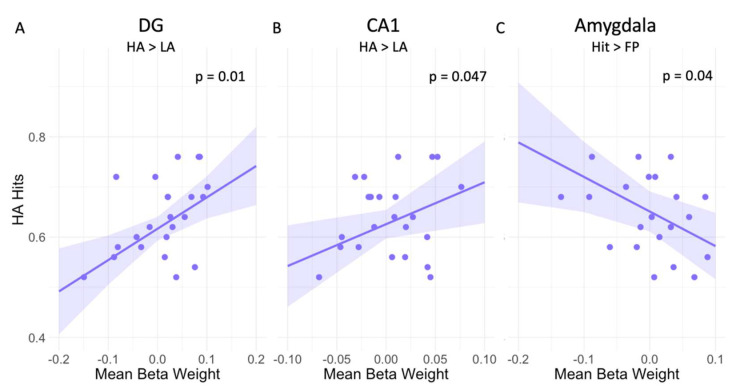
Association between mean ROI activity and memory performance under high arousal. Higher mean activity during high arousal compared to low arousal trials in the (**A**) DG is related to a higher proportion of hits (**B**) CA1 is related to a higher proportion of hits. (**C**) Greater deactivation in the amygdala for Hits compared to FP is associated with more high arousal Hits. Figures include the unadjusted *p*-values and colored bands representing the 95% CI. Abbreviations: CA = Cornu Ammonis, DG = dentate gyrus, Hit= correct answers, FP = false positives, HA = high arousal, LA = low arousal.

Upon investigating the effect of arousal on memory performance (Table 2), we observed that higher DG activity during HA trials compared to LA trials was related to a higher proportion of HA Hits (at trend level after multiple comparison correction, p_FDR_ = 0.05; Figure 3A). Additionally, higher activity in the CA1 during HA versus LA trials was related to a higher proportion of HA Hits (this effect did not survive correction for multiple comparisons, p_FDR_ = 0.12; Figure 3B). No other associations were observed for the amygdala, CA3, and LC (Table 2). Additionally, we found no associations between memory performance and activity in any of the ROIs for the other contrasts (Table 2).

Focusing on the effect of memory performance under different levels of arousal, we observed that better memory performance was related to greater amygdala deactivation during HA Hits compared to HA FP (this effect did not survive correction for multiple comparisons, p_FDR_ = 0.19; Figure 3C). We observed no other associations between memory performance and activity in the CA1, CA3 DG and LC for the contrast HA Hits > HA FP (Table 2). Additionally, no associations were observed for any of the ROIs for the other contrasts (Table 2).

### 3.2. Worry Modulates the Relationship between LC Activation and Mnemonic Discrimination Performance

**Figure 4:**
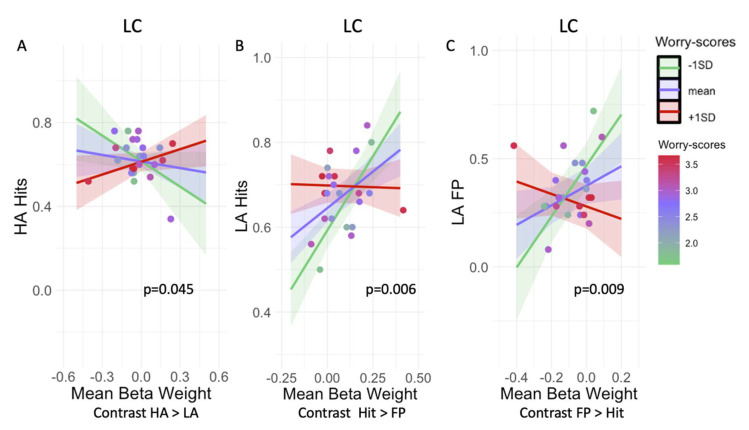
Visualization of the interaction between worry and LC activation on memory performance. (**A**) lower LC activity under high arousal is associated with higher hits for participants with a low compared to a high level of worry (**B**) Under low arousal higher LC activity for Hit trials than FP is associated with a higher proportion of hits in participants with low compared to high levels of worry (**C**) for participants with low compared to a high level of worry less deactivation of the LC is associated with more FP under low arousal. For visualization purposes the continuous variable worry is depicted as mean and +/− 1SD, as analyses were done with worry and activation as continuous variables (see Table 3). The color of the dots represents the continuous worry score of the participants. Abbreviations: LC = locus coeruleus, FP = false positives, HA = high arousal, LA = low arousal.

Investigating whether arousal modulates the association between worry and memory performance for the contrasts HA Hits > LA Hits (Table 3), we found that lower LC activity was associated with higher Hits during HA only for participants with low levels of worry compared to those with high levels of worry (p_FDR_ = 0.23; Figure 4A). Worry did not modify the relationships between hits and activity during this contrast for any of the other ROIs (amygdala, CA1, CA3, and DG).

Higher LC activity during Hit versus FP trials was associated with a higher proportion of Hits in participants with low compared to high levels of worry (p_FDR_ = 0.03; Figure 4B). No other associations for worry by ROI on memory performance for the amygdala, CA1, CA3, and DG were observed for this contrast (Table 3).

For the contrasts LA FP > LA Hit, we observed that in participants with lower worries, less deactivation of the LC was associated with more FP under low arousal in contrast to participants with high levels of worry (p_FDR_ = 0.045; Figure 4C). We observed no other associations for any of the ROIs for the other contrasts (Table 3).

We also checked the influence of depression score as a covariate in models 2 and 3; however, this did not change any of the interpretations of the results. Considering the relatively small sample size, we reported the more parsimonious model.

## 4. Discussion

**Figure 5:**
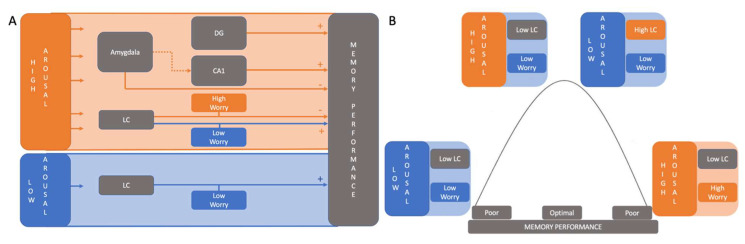
Schematic representation of the interactions and interpretations of the results in our paper. (**A**) High arousal effects are represented in orange. Low arousal effects are represented in blue. + represents higher ROI activation that is related to an improvement in memory performance. – represents a lower memory performance related to higher ROI activation. The dotted line depicts a relation we did not test but hypothesize based on previous literature. (**B**) Represents the model we hypothesize in which for optimal memory performance one source of arousal (high arousal stimuli or LC activation) leads to optimal performance which two sources of arousal push too far and leads to poor memory performance.

The current study examined whether worry, a commonly reported complaint in older adults and linked to a risk for later cognitive decline, changes the relationship between memory and neuronal activity patterns during an emotional memory task in older individuals. Consistent with previous work [61] we observed that under high arousal, older adults made more FP during this memory discrimination task. Additionally, higher LC activity was correlated with better memory performance in individuals with low worries, only if the context was under low arousal, suggesting that optimal memory performance requires a careful balance of NA-related activation (Figure 5). We did not observe a similar effect for individuals with high levels of worry. Our results suggest that in individuals with worry the noradrenergic system could possibly be an important part of the underlying mechanisms of lower memory functioning. The LC could therefore be a good target to further investigate future memory decline within a high-risk adult population.

Consistent with previous literature [18], a higher proportion of hits was facilitated by higher activity in the DG under high arousal. The DG does not only play an important role in mnemonic discrimination performance, but it also receives projections from the LC and the amygdala [2], which further supports its important role in accurate mnemonic discrimination performance under high arousal. We did not find a direct association between the LC and the amygdala and greater memory performance under high arousal. Additionally, we observed that (at trend-level) higher activity in the CA1 under high arousal was associated with a higher proportion of hits. This involvement of the CA1 under high arousal is consistent with the direct projections it receives from the amygdala [62], which are thought to modulate the strength of emotional memories [63]. In addition, in individuals with transient CA1 lesions, mnemonic discrimination performance was initially impaired but fully restored after the lesion was resolved [64], highlighting that the CA1 may play a role in mnemonic discrimination performance. In contrast, under high arousal, a higher deactivation of the amygdala during encoding was associated with a higher proportion of hits. This might indicate that participants who can suppress amygdala modulation under high arousal can learn more effectively. Although the activity of the amygdala is thought to promote better encoding of emotional material, earlier research has shown specifically for older adults that higher activity in the amygdala under high arousal was associated with false alarms [18]. Altogether, this suggests that in older adults, additional activity in the amygdala during a challenging memory task might drive over-generalization instead of successful pattern separation.

In line with our expectations, we observed that under low arousal, participants with a low level of worry benefitted from a higher LC activity resulting in better mnemonic discrimination performance. In contrast, under high arousal higher LC activity was associated with lower mnemonic discrimination performance in participants with low levels of worry. Under both high and low arousal for highly worried participants, the LC activity does not seem to modulate memory performance. This could suggest that, in individuals with a higher level of worry, the ability of the LC to efficiently modulate memory performance seems to be compromised, while in individuals with low worry there might be an optimal arousal level depending on LC activity and the arousal induced by the task. Although speculative, this could fit within the canonical Yerkes-Dodson relationship [5], according to which arousal and performance are carefully balanced. We suggest that for optimal mnemonic memory performance there is also a need for an optimal level of involvement of the NA-system. Under lower levels of arousal higher LC activity might be needed to achieve similar levels of optimal memory performance as achieved under higher arousal. However, to really understand the Yerkes-Dodson relationship between LC activity and memory performance, and the potential modification by worry, additional measurements sensitive to the disbalance between tonic and phasic activity of the LC such as pupil measurements are needed, as BOLD cannot reveal information about phasic activity [5] to us. This could provide us with further information on how the Yerkes-Dodson relationship between LC activity and memory performance changes under high worry and high arousal in older adults.

In contrast to our expectations and earlier research [4,18], we did not observe an enhancing effect of high arousal on memory performance. We observed an increase in FP under high arousal, indicating that mnemonic discrimination might be breaking down under high arousal as similar items were more often incorrectly classified as old. Although the enhancing effects of encoding stimuli in a high arousing context have been shown to benefit recollection [1,2,44,65], there is also evidence for higher FP under high arousal conditions [61]. A possible explanation could lie in an emotion-induced memory trade-off in which the gist of the content is remembered but details are often forgotten [15,66]. These effects usually become more pronounced after longer consolidation periods than those allowed for in our study [67]. However, worry is associated with a lower emotional regulation capacity [32]. Therefore, our selection of participants along the worry continuum may explain why our findings deviate from the literature. This might suggest a heightened vulnerability to the negative effects of additional arousal in older adults with a broad range of worries. Supporting this suggestion, we observed that only under low arousal was there a trend-level positive relation between worry about memory functioning and the proportion of hits, while no relation was found for high arousal trials. While we need to be cautious in interpreting this result, this could suggest that under low arousal conditions participants with a high level of worry about memory functioning can still allocate additional resources for optimal performance when confronted with a challenging memory task [68]. We did not observe a similar result under high arousing conditions, suggesting that under additional arousal either for individuals with high worry the process of additional resource allocation is dysfunctional and therefore does not result in better memory performance or that all resources might already be depleted by the additionally added arousal.

There are several limitations to our study. Imaging activity of the human LC using fMRI is challenging due to its small size and sensitivity to physiological noise. To tackle this, we visualized the LC at an individual level with a high-resolution in-house developed MT-weighted technique [61,69] from which we extracted a study-specific LC template. Additionally, confounding signals stemming from the LC’s proximity to the fourth ventricle were removed. However, future studies should consider acquiring additional physiological data to correct for increased physiological noise associated with high arousal tasks. Although we averaged over the left and right hemisphere, future studies should consider further exploring a disbalance in activity between the two LC’s and the hemisphere, as this has been shown to be related to cognitive functioning and could thereby further support our results [70,71]. Furthermore, our sample size was relatively small, which might have contributed to some of our effects not surviving multiple comparison corrections, and reproducing these results with a larger sample size will be important. Finally, our small sample size did not allow us to further examine whether arousal directly modulated the association between worry and memory performance.

## 5. Conclusions

In conclusion, our results show that in a group of cognitively healthy older adults with various levels of worry, a context of high arousal affects mnemonic discrimination performance, resulting in higher FP compared to low arousal. Additionally, our results suggest that there might be an optimal involvement of the NA-system for optimal memory discrimination performance, as we observed for people with low levels of worry that under lower levels of arousal higher LC activity might be needed to achieve similar levels of optimal memory performance as achieved under higher arousal when LC activity remained lower. These findings may provide information on who may benefit most from preventive or NA-targeted interventions. However, more research is needed in larger populations to extend these findings to both structural and functional longitudinal changes in the NA-system in groups with a broad range of worry.

## Figures and Tables

**Table 1:** Characteristics of Participants.

	Mean (SD)
N	23
Age (years)	67.1 (4.9)
Sex n (% female)	47.8%
Education level	4.3 (1.6)
MMSE (score)	29.3 (0.8)
VLT-total (nr words)	46.7 (8.5)
VLT-delayed (nr words)	9.2 (2.5)
HDRS (score)	3.8 (3.6)
MIA anxiety (score)	2.8 (0.6)
High Arousal Hits (score)	0.6 (0.08)
Low Arousal Hits (score)	0.7 (0.08)
High Arousal FP (score)	0.5 (0.2)
Low Arousal FP (score)	0.4 (0.2)
High Arousal FN (score)	0.2 (0.1)
Low Arousal FN (score)	0.2 (0.1)

Education level was determined on an 8-point scale according to the standard Dutch classification system comparable to International standard classification of Education [60] MMSE = Mini Mental State Exame; VLT = verbal word learning task; HDRS = Hamilton Depression Rating Score; MIA = Meta-memory In Adulthood; FP = False Positives; FN = False Negatives.

**Table 2:** Association between arousal or memory-related activity in our ROIs and memory performance.

	Est.	*t*	*p*	P (FDR)
Arousal Contrast: Hit HA-LA				
Amygdala	0.03	0.06	0.95	0.95
CA1	0.84	2.08	**0.047**	0.12
CA3	0.31	1.06	0.30	0.50
DG	0.63	2.82	**0.01**	0.05
LC	0.04	0.33	0.74	0.93
Arousal Contrast: Hit LA-HA				
Amygdala	−0.22	−0.57	0.57	0.71
CA1	−0.85	−1.43	0.18	0.71
CA3	−0.07	−0.20	0.84	0.84
DG	−0.24	−0.66	0.51	0.71
LC	−0.09	−0.59	0.56	0.71
Arousal Contrast: FP HA-LA				
Amygdala	0.54	1.39	0.18	0.40
CA1	−0.12	−0.22	0.83	0.83
CA3	−0.10	−0.33	0.74	0.83
DG	−0.41	−1.41	0.17	0.40
LC	−0.29	−1.21	0.24	0.40
Arousal Contrast: FP LA-HA				
Amygdala	−0.34	−0.97	0.33	0.48
CA1	−0.27	−0.53	0.59	0.59
CA3	0.23	0.86	0.38	0.48
DG	0.28	0.91	0.37	0.48
LC	0.28	1.04	0.31	0.48
Memory Contrast: HA Hit-FP				
Amygdala	−0.69	−2.22	**0.037**	0.19
CA1	−0.06	−0.17	0.87	0.87
CA3	−0.35	−1.49	0.15	0.32
DG	−0.32	−1.35	0.19	0.32
LC	−0.10	−0.55	0.59	0.74
Memory Contrast: LA Hit-FP				
Amygdala	−0.16	−0.58	0.58	0.73
CA1	−0.36	−1.03	0.30	0.73
CA3	−0.03	−0.15	0.88	0.88
DG	0.13	0.56	0.58	0.73
LC	0.13	0.74	0.47	0.73
Memory Contrast: HA FP-Hit				
Amygdala	−0.57	−0.98	0.36	0.50
CA1	−0.60	−0.88	0.40	0.50
CA3	−0.71	−1.40	0.18	0.45
DG	−0.82	−1.65	0.12	0.45
LC	−0.10	−0.32	0.75	0.75
Memory Contrast: LA FP-Hit				
Amygdala	0.17	0.34	0.74	0.74
CA1	−0.21	−0.35	0.73	0.74
CA3	0.35	1.24	0.23	0.47
DG	0.52	1.57	0.13	0.47
LC	0.33	1.25	0.28	0.47

Note. Robust regression models are adjusted for age, and sex. Beta coefficients are unstandardized. Abbreviations: CA = Cornu Ammonis, DG = dentate gyrus, LC = locus coeruleus, FP = false positives, HA = high arousal, LA = low arousal, Est = estimate (i.e., the coefficient), P(FDR) = False Discovery Rate corrected *p*-value. *p*-values < 0.05 formatted in bold.

**Table 3:** Effect modification of worry on the association between activity (memory and arousal contrasts) and memory performance.

	Est.	t	*p*	P (FDR)
Arousal Contrast: Hit HA-LA				
Amygdala	0.32	0.46	0.65	0.81
CA1	1.31	1.40	0.19	0.35
CA3	0.83	1.28	0.21	0.35
DG	0.03	0.07	0.94	0.94
LC	0.52	2.1	**0.045**	0.23
Arousal Contrast: Hit LA-HA				
Amygdala	0.02	0.03	0.98	0.98
CA1	1.01	0.87	0.40	0.92
CA3	−0.57	−0.89	0.38	0.92
DG	−0.08	−0.13	0.90	0.98
LC	−0.21	−0.61	0.55	0.92
Arousal Contrast: FP HA-LA				
Amygdala	0.94	1.20	0.24	0.99
CA1	0.02	0.02	0.99	0.99
CA3	−0.11	−0.21	0.84	0.99
DG	0.39	0.62	0.55	0.99
LC	−0.11	−0.23	0.82	0.99
Arousal Contrast: FP LA-HA				
Amygdala	−0.21	−0.26	0.79	0.99
CA1	0.50	0.49	0.63	0.99
CA3	−0.05	−0.11	0.91	0.99
DG	−0.28	−0.45	0.66	0.99
LC	0.01	0.01	0.99	0.99
Memory Contrast: HA Hit-FP				
Amygdala	1.30	1.76	0.10	0.50
CA1	1.06	1.23	0.23	0.58
CA3	0.20	0.48	0.64	0.69
DG	0.19	0.40	0.69	0.69
LC	0.30	0.78	0.44	0.69
Memory Contrast: LA Hit-FP				
Amygdala	0.38	0.60	0.55	0.69
CA1	0.30	0.65	0.53	0.69
CA3	0.15	0.50	0.62	0.69
DG	−0.18	−0.41	0.69	0.69
LC	−0.61	−3.13	**0.006**	**0.03**
Memory Contrast: HA FP-Hit				
Amygdala	1.21	0.78	0.44	0.69
CA1	0.83	0.62	0.55	0.69
CA3	−0.04	−0.04	0.97	0.97
DG	0.77	0.85	0.43	0.69
LC	0.68	1.18	0.26	0.69
Memory Contrast: LA FP-Hit				
Amygdala	−0.80	−0.67	0.51	0.82
CA1	0.22	0.24	0.81	0.82
CA3	−0.11	−0.23	0.82	0.82
DG	−0.77	−1.07	0.30	0.75
LC	−1.25	−2.92	**0.009**	**0.045**

Note. Robust regression models for the interaction between activity and worry on memory performance, adjusted for age and sex. Unstandardized Beta coefficients represent the interaction term between worry and ROI activity. Abbreviations: CA = Cornu Ammonis, DG = dentate gyrus, LC = locus coeruleus, FP = false positives, HA = high arousal, LA = low arousal Est = estimate (i.e., the coefficient), P(FDR) = False Discovery Rate corrected *p*-value. *p*-values < 0.05 formatted in bold.

## Data Availability

The data presented in this study are available on request from the corresponding author.

## Supplementary Materials

The following supporting information can be downloaded at: https://www.mdpi.com/article/10.3390/brainsci12030381/s1, S1: Latent class analysis, Table S1: Latent Class model fit, Figure S1: Trajectory of MIA- worry score over time of the different latent classes, Figure S2: The association between Worry and Depression score, Figure S3: Violin plot showing the distribution of the mean activation per ROI divided by arousal level, Figure S4: Violin plots showing the distribution of the mean activation per ROI divided by hits and FP. Figure S5: The scan of the LC in template space.
