# Supplementary material for: Worry Modifies the Relationship between Locus Coeruleus Activity and Emotional Mnemonic Discrimination
Source: Brain Sci. 2022 Mar 12;12(3):381. doi: 10.3390/brainsci12030381 (PMC8946181; doi:10.3390/brainsci12030381)
Supplement: Supplementary file 1 [file brainsci-12-00381-s001.zip › brainsci-1613970-supplementary.pdf]

Supplementary Data

S1 Latent class analysis

From the MAAS cohort 800 participants between the age of 35-70 completed the Metamemory In Adulthood (MIA) questionnaire for at least 3 measurements over a period of 9 years. A latent class analysis was performed to determine the trajectories of worry about memory functioning measured with the MIA-anxiety subscale. The latent class analysis was performed for 2, 3, 4 and 5 classes (see table S1). The latent class analysis model with the most optimal fit represented by both a smaller AIC and BIC without creating fragmentation resulting in small groups revealed 4 classes (Figure S1): Class 1 demonstrated increasing levels of worry about cognitive performance over time (n=23); Class 2 Stable high worried group (n=284); Class 3 demonstrated decreasing levels of worry about cognitive performance over time (n=15); Class 4 stable low worries about cognitive function n=478). From class two and class four participants were contacted for inclusion in the current study.

Table S1. Latent Class model fit

	**2 Classes**	**3 Classes**	**4 Classes**	**5 Classes**
AIC	4138.6	4120.54	4107	4104.15
BIC	4176.07	4172.07	4172	4183.79

AIC = Akaike information criterion, BIC = Bayesian information criterion

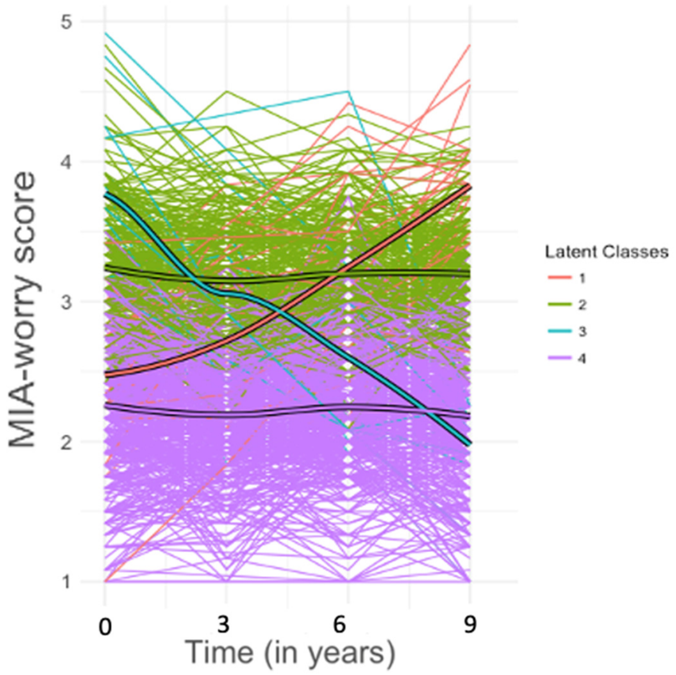

Figure S1 Trajectory of MIA- worry score over time of the different latent classes

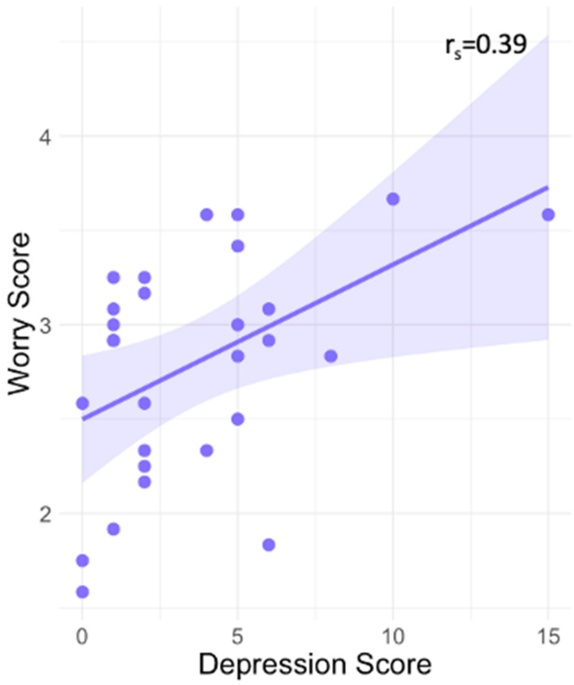

Figure S2 The association between Worry and Depression score, showed a positive Spearman correlation between Worry and the depression score r_s=0.39, p=0.04

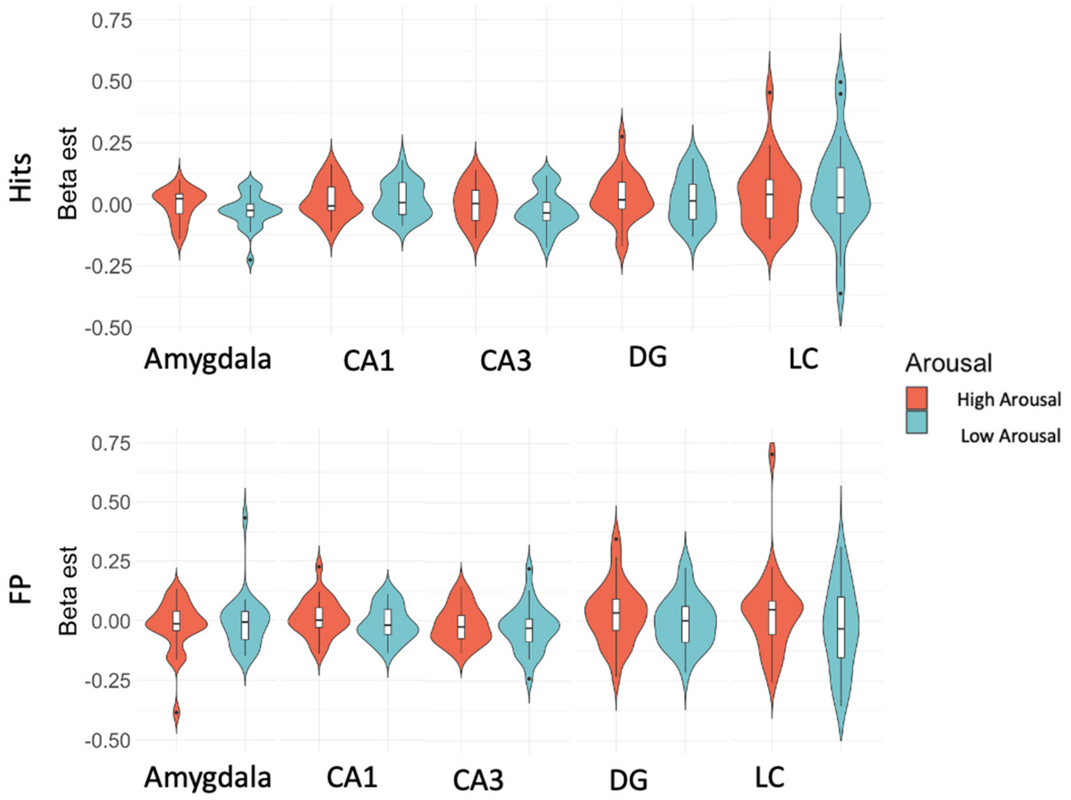

Figure S3 Violin plot showing the distribution of the mean activation per ROI divided by arousal level. The boxplots shows the median and upper and lower quartiles. No significant differences were observed for any of the ROI's $p<0.05$.

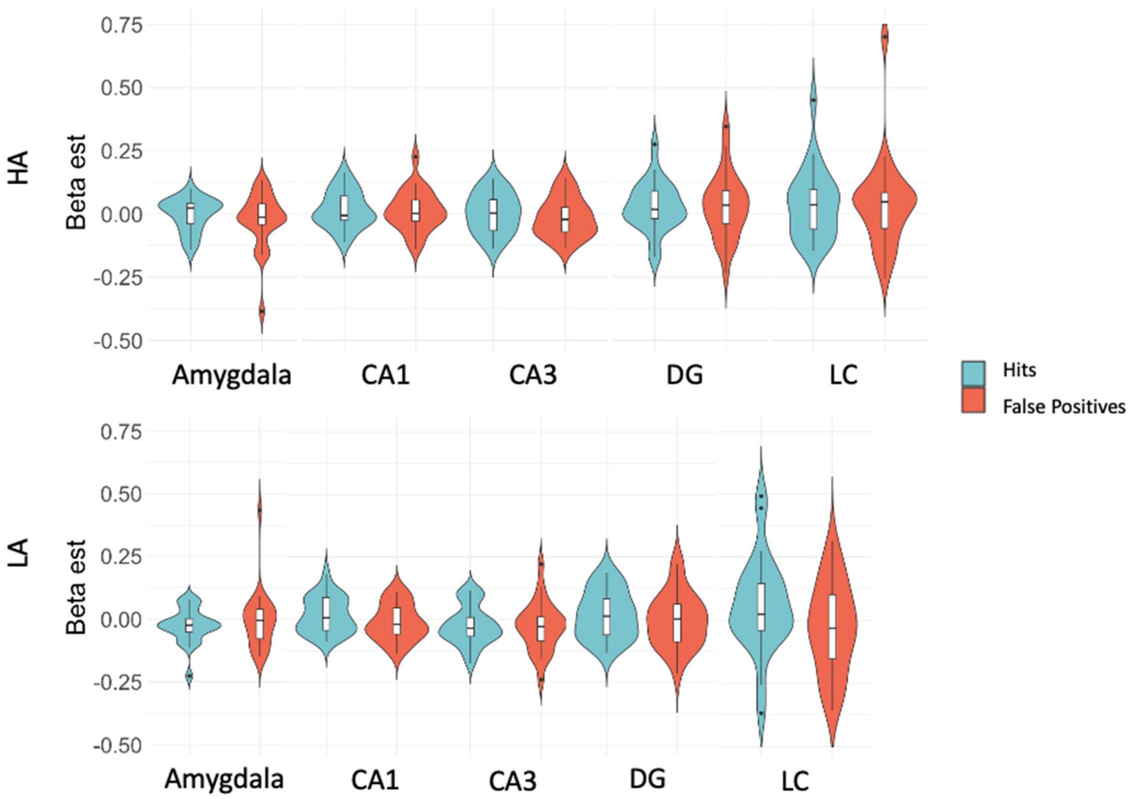

Figure S4 Violin plots showing the distribution of the mean activation per ROI divided by hits and FP. The boxplots shows the median and upper and lower quartiles. No significant differences were observed for any of the ROI's p<0.05.

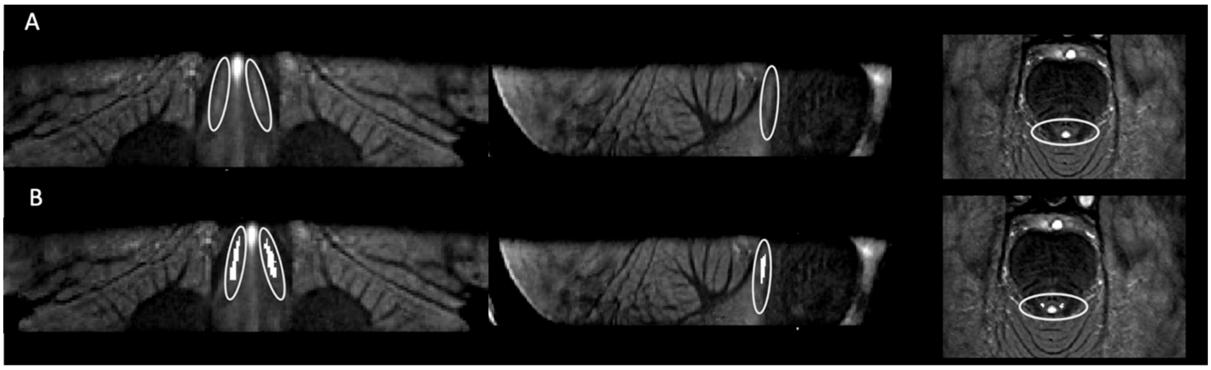

Figure S5 The scan of the LC in template space A) without and B) with the LC-ROI superimposed
